# Long-Term Prognosis of Patients with Polypoidal Choroidal Vasculopathy Treated with Photodynamic Therapy

**DOI:** 10.3390/jcm12144707

**Published:** 2023-07-16

**Authors:** Yukinori Sakaeda, Aki Kato, Soichiro Kuwayama, Shuichiro Hirahara, Norihiro Suzuki, Yuichiro Ogura, Yoko Nakazawa, Tsutomu Yasukawa

**Affiliations:** 1 Department of Ophthalmology and Visual Science, Nagoya City University Graduate School of Medical Sciences, 1 Kawasumi, Mizuho-cho, Mizuho-ku, Nagoya 467-8601, Japan; 2 Department of Ophthalmology, Nagoya City University, West Medical Center, 1-1-1 Hirate-cho, Kita-ku, Nagoya 462-8508, Japan

**Keywords:** age-related macular degeneration, anti-vascular endothelial growth factor therapy, photodynamic therapy, polypoidal choroidal vasculopathy

## Abstract

We evaluated the long-term prognosis of the eyes of patients with polypoidal choroidal vasculopathy (PCV) treated with photodynamic therapy (PDT). In total, 60 eyes of 57 patients diagnosed with PCV and treated with PDT were reviewed retrospectively in real-world settings. The best-corrected visual acuity (BCVA), central retinal thickness (CRT), anatomical findings (vision-threatening findings), and treatment history were assessed. In total, 38 eyes underwent PDT as the initial treatment (initial PDT group) and 22 eyes underwent PDT as a rescue treatment (rescue PDT group). In the initial PDT group, 11 eyes (29%) did not require additional therapy throughout the observation period and maintained good BCVA. A total of 27 eyes (71%) underwent additional treatments and the mean BCVA was only stabilized for 2 years; thereafter, decreased vision occurred even with additional treatments. In the rescue PDT group, 22 eyes (95%) required additional treatment. Hard exudate, serous pigment epithelial detachment, and the total vision-threatening score were related to worse BCVA. Initial PDT may be effective in about 30% of cases with preservation of good vision and no need for additional treatment. However, patients with received rescue PDT needed additional treatment in most cases and the vision decreased in many cases.

## 1. Introduction

Neovascular age-related macular degeneration (NV-AMD) is a major cause of visual loss in elderly individuals in developed countries and the incidence in Japan has increased in recent years [1]. Polypoidal choroidal vasculopathy (PCV), a subtype of NV-AMD, is characterized by polypoidal lesions with or without branching vascular networks under the retinal pigment epithelium; it causes retinal pigment epithelial detachments (PEDs), serous retinal detachment (SRDs), and hemorrhages in multiple retinal layers [2,3]. PCV is particularly common in Asians [4,5,6].

Photodynamic therapy (PDT) with verteporfin (Visudyne^®^, Novartis AG, Basel, Switzerland) uses low-energy wavelengths and thus does not damage the photoreceptors, allowing treatment of macular lesions. The laser activation of intravenously administered verteporfin produces a photochemical reaction rather than a photothermal one, resulting in the generation of free radicals that induce subsequent endothelial damage, platelet-activation localized thrombosis, and the occlusion of choroidal neovascular membranes. PDT is predominantly effective for treating classic choroidal neovascularization [7,8]. The Japanese Age-Related Macular Degeneration Trial (JAT study) Study Group also demonstrated the usefulness of PDT [9], which was approved in 2004 in Japan. Since the intravitreal injection of the anti-vascular endothelial growth factor (VEGF) agent, ranibizumab (IVR) (Lucentis^®^, Genentech Inc., South San Francisco, CA, USA), was approved for NV-AMD, the first-choice treatment for NV-AMD has been anti-VEGF therapies [10]. However, based on some clinical reports indicating that PDT is useful for PCV, prospective studies of PDT in combination with anti-VEGF therapy, i.e., the EVEREST II study [11,12] and the PLANET study [13], were conducted. The patients in both studies were Asians with PCV.

The EVEREST II trial [11,12] reported that with combination therapy of the initial PDT application and ranibizumab, visual acuity (VA) improved, polypoidal lesions regressed, and the number of injections decreased compared to ranibizumab monotherapy. However, the PLANET study [13] reported no significant difference in VA and exudative changes when PDT was combined with aflibercept (Eylea^®^, Regeneron Pharmaceuticals, Tarrytown, NY, USA) for rescue purposes.

Few reports have evaluated the long-term VA, anatomic findings, or additional treatment in patients treated with PDT monotherapy or PDT/anti-VEGF combination therapy for PCV. The current study evaluated the long-term prognosis of eyes with PCV treated with PDT after approval of anti-VEGF therapy in real-world settings.

## 2. Materials and Methods

### 2.1. Study Design and Ethics

This was a retrospective single-center observational study conducted in Nagoya City University Hospital (UMIN registration number, UMIN000046415). The institutional review board of Nagoya City University Graduate School of Medical Science approved the study protocol which was conducted in compliance with the ethical guidelines of the Declaration of Helsinki. The requirement for informed consent was waived because of the retrospective observational nature of the study.

### 2.2. Patients

The study included 60 eyes of 57 patients (42 men and 15 women) who were diagnosed with PCV at Nagoya City University Hospital between April 2009 and December 2019, underwent PDT, and were followed for at least 1 year. The exclusion criteria were eyes for which the necessary data were lacking or eyes with a history of other vitreoretinal diseases such as retinal detachment, diabetic retinopathy, retinal vein occlusion, and uveitis.

### 2.3. Research and Analysis

The medical data assessed included the patient’s age at the start of treatment of PCV, follow-up periods, previous treatment history, and the timing and frequency of additional treatments (anti-VEGF injections: IVR, aflibercept, or PDT). The ophthalmologic examination, including the best-corrected VA (BCVA) in decimal units and the central retinal thickness (CRT) measured by optical coherence tomography (OCT) was conducted at the start of treatment with PCV, just before PDT, every year after PDT to a maximum of 5 years, and at the final visit. The analysis of the VA was performed by converting the values from decimal units to a logarithm of the minimum angle of resolution (logMAR) units.

The greatest linear dimension (GLD) and the presence of subretinal fluid (SRF) intraretinal fluid (IRF), serous retinal PED, subretinal hemorrhage (SRH), hard exudates (HE), and subretinal hyperreflective material (SHRM) before the initial PDT were evaluated. At the final visit, fibrovascular PED, fibrosis, and atrophy including the fovea were evaluated.

The patients were divided into two groups for analysis: those who received PDT as an initial treatment (the initial PDT group who underwent a sub-Tenon triamcinolone acetonide injection (STTA)/PDT application with/without anti-VEGF therapy in the treatment-naïve eyes) and those who received PDT during treatment (the rescue PDT group who underwent STTA/PDT with/without anti-VEGF therapy in eyes with frequent recurrences or those refractory to anti-VEGF therapy). The visual outcomes were also evaluated by further dividing the patients into 3 subgroups: (1) rescue within 1 year with additional treatment within a year; (2) rescue after 1 year with additional treatment during follow-up; and (3) no additional treatment.

The vision-threatening score was defined as a total score of 1 point for “yes” and 0 points for “no” for the presence of SRF, IRF, serous PED, SRH, HE, and SHRM; 1 point for a GLD of 4000 µm or more; and 0 points for GLD less than 4000 µm.

### 2.4. Statistical Analysis

The differences in the proportions of patients who required additional treatment were analyzed by the Kaplan–Meier survival time using the log-rank test. The mean changes in the BCVAs and CRTs at each time point were compared using analysis of variance (ANOVA) (Bonferroni’s test). Dunnett’s test was used to analyze differences in the vision-threatening score based on the BCVA at the final visit. Pearson’s correlation coefficient or Spearman’s rank correlation coefficient was used to analyze the risk factor effect of the final BCVA. A value of *p* < 0.05 was considered significant. Microsoft Excel software (Microsoft Corporation, Redmond, WA, USA) or IBM SPSS Statistics (International Business Machines Corporation, Armonk, NY, USA) was used for the statistical analyses.

## 3. Results

### 3.1. Patient Characteristics

The patient characteristics in the initial PDT group and the rescue PDT group are shown in Table 1. In total, 38 eyes of 35 patients underwent PDT as the initial treatment. Thirty-four eyes underwent PDT and received STTA, five of which also received anti-VEGF at the same time. In four cases, STTA was not used because the supply from the supplier was interrupted from May to June 2009. In total, 22 eyes of 22 patients underwent PDT as a rescue treatment. The mean time from the initial treatment to initiation of PDT was 1.6 years and the mean number of anti-VEGF injections during that time was 7.3 times. The anti-VEGF therapy regimens consisted of 19 eyes on the pro re nata (PRN) regimen, 2 eyes on the treat-and-extend (TAE) regimen, and 1 eye that was switched from the PRN regimen to the TAE regimen. PDT was applied in 13 eyes because of frequent recurrences and the inability to extend the treatment interval, in 5 eyes because of the refractoriness to treatment and failure to improve the exudative changes, and in 4 eyes because the patient requested it. All cases underwent PDT combined with STTA and eight also received anti-VEGF therapy at the same time.

### 3.2. Additional Treatments

Table 2 shows the additional treatments in each group after the first PDT treatment. In the initial PDT group, 17 eyes (45%) required additional treatment within 1 year (mean number of anti-VEGF injections, 1.6; mean number of PDT applications, 0.30) and 27 eyes (71%) required additional treatments during the follow-up period (6.2 years) (mean number of anti-VEGF injections, 7.9; mean number of PDT applications, 0.26). In the rescue PDT group, 13 eyes (59%) required additional treatments within 1 year (mean anti-VEGF injections, 1.9; mean number of PDT applications, 0.15) and 21 eyes (95%) required additional treatment during the follow-up period (4.3 years) (mean number of anti-VEGF injections, 6.7; mean number of PDT applications, 0.32). All additional PDT applications were performed combined with STTA.

**Figure 1:**
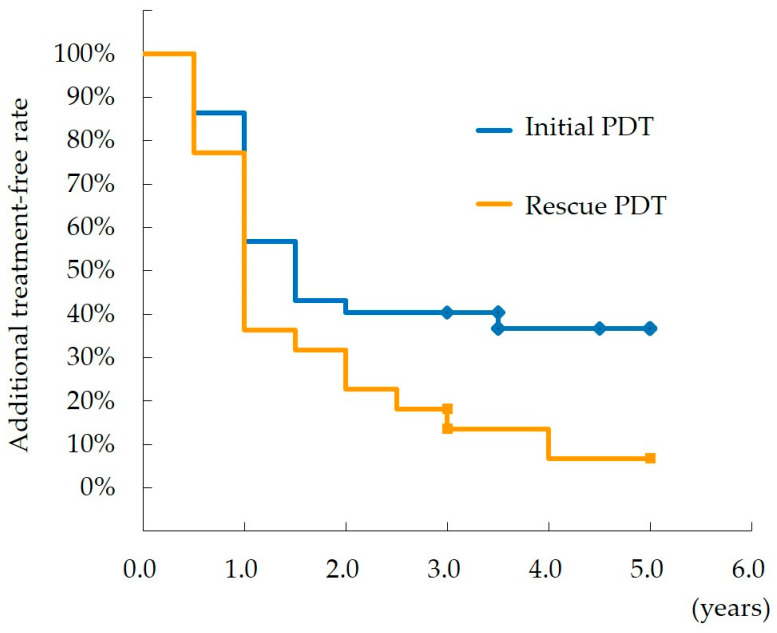
Times of additional treatments. In the initial PDT group, 37% of patients did not require additional treatments at year 5, while in the rescue PDT group, significantly fewer patients (9%) did not require additional treatments (Kaplan–Meier; log-rank test, *p* < 0.05).

The times of the additional treatment are shown in Figure 1. In the initial PDT group, 55% of patients did not require additional treatment at year 1 while 29% did at the final visit; in the rescue PDT group, 41% of patients did not require additional treatment at year 1 and 5% did at the final visit. In the initial PDT group, significantly more patients did not require additional treatment (37%) compared to the rescue PDT group (9%) at year 5 (Kaplan–Meier; log-rank test, *p* < 0.05).

### 3.3. Changes in BCVA and CRT

**Figure 2:**
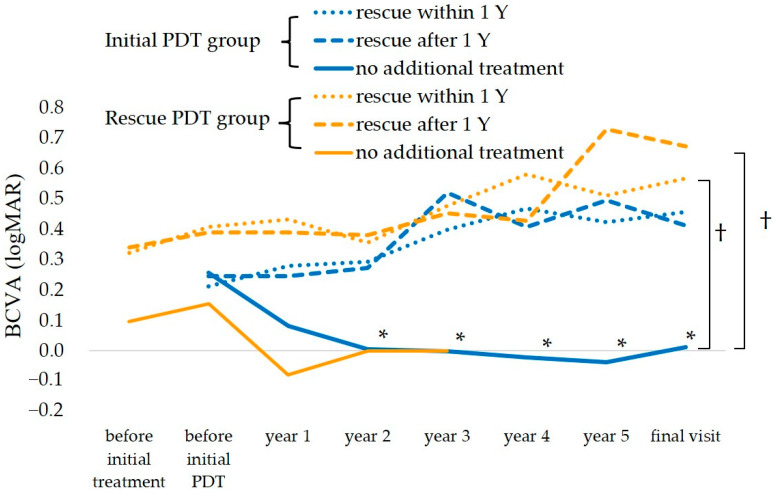
Changes in BCVA. The initial PDT (no additional treatment) group (solid blue line) had significantly improved BCVA after the second year which was maintained until the final visit; this group also had significantly better final BCVA than the rescue PDT (rescue within 1 year) group or rescue PDT (rescue after 1 year) group (dotted orange lines). * *p* < 0.01, ^†^ *p* < 0.01, ANOVA (Bonferroni’s test). Y = year.

In the initial PDT group, the BCVA changed from 0.26 before PDT to 0.22, 0.20, and 0.36 at years 1, 2, and 5, respectively. In the rescue PDT group, the BCVA changed from 0.32 before treatment to 0.44, 0.39, 0.35, and 0.62 before initial PDT and at years 1, 2, and 5, respectively. The mean changes in the BCVA are shown in Figure 2. In the initial PDT group with no additional treatment, the BCVA changed from 0.26 before treatment to 0.08 at 1 year, 0.01 at 2 years, −0.04 at 5 years, and 0.01 at the final visit; the BCVA improved significantly compared to before treatment at every time point (*p* < 0.01, Bonferroni’s test). The initial PDT group with no additional treatment also had significantly better final BCVA than the rescue PDT (rescue within one year) group and rescue PDT (rescue after one year) group (*p* < 0.01, Bonferroni’s test). The other groups showed no significant VA changes during follow-up.

**Figure 3:**
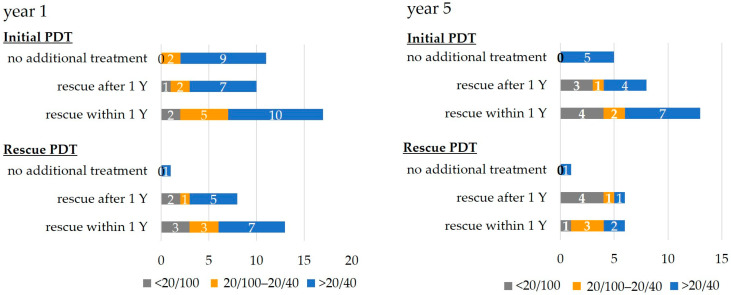
Proportion of BCVA at 1 and 5 years. In the initial PDT group, good BCVA was maintained in more than half of the eyes at both 1 and 5 years. In the rescue PDT group, half of the patients had good vision at 1 year but the number of patients who maintained good BCVA at 5 years decreased. Y = year.

The number of patients at each BCVA level at years one and five are shown in Figure 3. In the initial PDT groups, good BCVA was maintained in more than half of the eyes at one year in all the subgroups, while at five years, in the no additional treatment group, five eyes maintained good BCVA. In the groups that needed additional treatment (rescue after one-year group or rescue within one-year group), half of the eyes maintained good BCVA. In the rescue PDT groups, good BCVA was maintained in more than half of the eyes at one year but the number of patients who maintained good BCVA at five years decreased.

**Figure 4:**
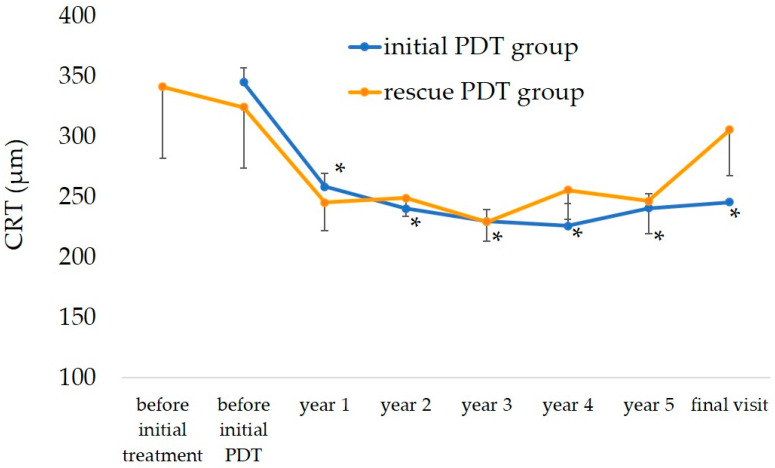
Changes in CRT. In the initial PDT group, the mean CRT improved significantly after the first year and was maintained until the final visit. In the rescue PDT group, the CRT tended to improve by the third year but gradually returned to the pre-initial PDT level from the fourth year. * *p* < 0.01 ANOVA (Bonferroni’s test) bar = ± standard error of the mean.

The changes in the CRT are shown in Figure 4. In the initial treatment group, the mean CRT decreased from 345 μm before treatment to 259 μm at 1 year, 240 μm at 2 years, 241 μm at 5 years, and 246 μm at the final visit. The CRT improved significantly compared to before treatment at all time points (*p* < 0.01, by Bonferroni’s test for all comparisons). In the rescue PDT group, the CRT changed from 342 μm before the initial treatment to 325 μm just before the initial PDT, 245 μm at 1 year, 250 μm at 2 years, 246 μm at 5 years, and 306 μm at the final visit. The CRT tended to decrease until the 5-year time point compared to just before the initial PDT but the change was not significant.

### 3.4. Distribution of Final BCVAs

**Figure 5:**
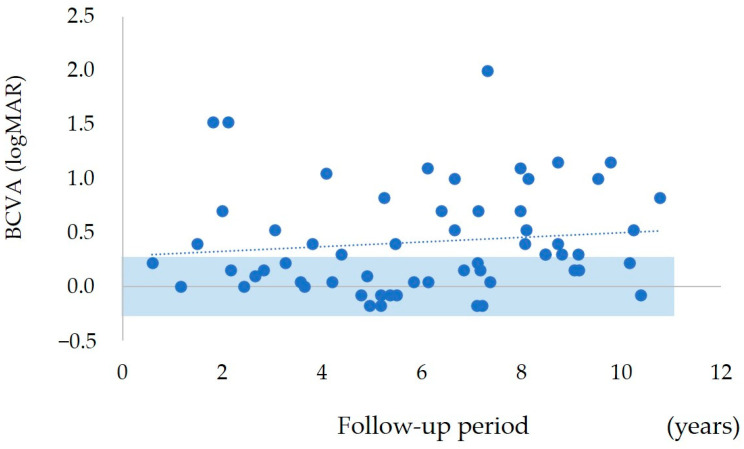
Distribution of the final BCVAs. The shaded area indicates the final BCVA is better than 0.22 logMAR. In total, 29 of the 60 eyes had a final BCVA of 0.22 or better and 18 of these patients had a follow-up period of 5 years or longer after the initial treatment.

The distribution of the final BCVA for all cases is shown in Figure 5. A total of 29 of the 60 eyes had a final BCVA of 0.22 or better in logMAR units; 18 of these eyes maintained good BCVA for more than 5 years after the initial treatment.

### 3.5. Score of Vision-Threatening Findings and Final BCVA

**Figure 6:**
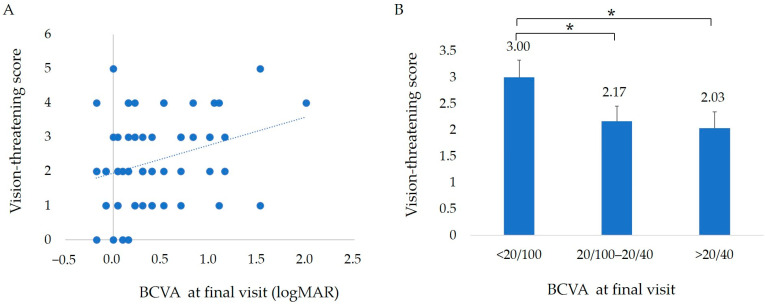
Score of vision-threatening findings and final BCVA. The vision-threatening score was defined as the total score of the presence of SRF, IRF, serous PED, SRH, HE, SHRM, and GLD of 4000 µm or more, each of which was considered to be 1 point. The score of the vision-threatening findings tended to be higher with the worsening of the final BCVA. (**A**) The mean score of the vision-threatening findings in the group with a final BCVA less than 20/100 was significantly higher than the group with a final BCVA of 20/100 to 20/40 and the group with a BCVA better than 20/40 was (**B**) (Dunnett’s test * *p* < 0.05).

The distributions of the vision-threatening score, defined as the total score of the SRF, IRF, serous PED, SRH, HE, SHRM, and GLD before the initial treatment and the BCVA at the final visit are shown in Figure 6A. The score tended to be higher with the worsening of the final BCVA; however, the difference did not reach statistical significance. The mean total scores classified by the BCVA at the final visit are shown in Figure 6B. The mean score for a BCVA of 20/100 or less was significantly higher than the group with a final BCVA of 20/100 to 20/40 and the group with a BCVA better than 20/40 (Dunnett’s test * *p* < 0.05).

### 3.6. Correlations between the Final VA and Each Parameter

Table 3 shows that the final logMAR BCVA was correlated negatively with the time until additional treatment and positively with the serous PED and hard exudates. No correlation was seen between the SRF, IRF, SRH, SHRM, and GLD. However, the total vision-threatening score was also correlated positively with the BCVA at a baseline, BCVA at year one, fibrosis, and atrophy at the final visit (*p* < 0.05, Pearson’s correlation coefficient, or Spearman’s rank correlation coefficient).

## 4. Discussion

The JAT study, the first PDT trial in Japan, reported that PDT improved mean VA [9,14]. The improvement in the mean VA with PDT in the JAT study differed from the TAP and VIP studies [7,8] in which patients only maintained a mean VA. This may be because the PCV responded well to PDT in a sub-analysis of the JAT study [14]. A later prospective Japanese study showed that PDT was more effective in patients with PCV than AMD [15].

In the current study, we evaluated the long-term prognosis of eyes with PCV treated with PDT after the approval of anti-VEGF therapy. In this study, more men than women were included. Epidemiologic studies [16] and previous reports [4] have shown that PCV is more common among men in Japan and the present results are consistent with these previous reports.

For patients in the initial PDT group, the mean BCVA was maintained and the mean CRT improved during the follow-up period. Of these patients, 55% did not require additional treatment within 1 year and 29% did not require treatment during the follow-up period. The patients who did not require additional treatment maintained good VA for five years. About half of the patients who required rescue treatment in the initial PDT group also maintained good VA. Regarding the additional treatment, the mean number of additional anti-VEGF injections and PDT were 7.9 and 0.26 during the entire follow-up period. Considering that the average follow-up after the initial PDT application was 5.4 years, anti-VEGF injections were administered only about once or twice annually, even in eyes that required additional treatment. The EVEREST II study [12] reported that the combination of initial PDT and IVR improved VA by 8.3 letters (Early Treatment of Diabetic Retinopathy Study: ETDRS) and CRT by 164 µm. IVR injections were administered 5.2 times at 12 months. Takahashi et al. [17] systematically reviewed the effectiveness of clinical treatments for NV, that is AMD in Japanese patients, and analyzed various parameters of typical AMD and PCV after one year of treatment. They reported that, in the group treated with anti-VEGF plus PDT, the BCVA changed from 0.51 to 0.32 (logMAR units), the CRT changed from 364 µm to 189 µm, and additional treatments with anti-VEGF was administered 3.2 times. In the PDT monotherapy group, the VA changed from 0.61 to 0.50 (logMAR units) and the CRT changed from 364 µm to 282 µm. In our study, the change in VA was 0.04, an improvement of about 2 letters of ETDRS, while the CRT improved by 86 µm. The number of additional treatments was 1.6; these results are similar to those of the EVEREST II study [12] and those reported by Takahashi et al. [17] in which the initial PDT application improved the VA and reduced the number of anti-VEGF injections. In this study, the initial PDT group included the patients who received PDT and anti-VEGF therapy simultaneously as an initial treatment. Several reports have shown the efficacy of the combination of PDT and anti-VEGF therapy [18,19,20]. However, further comparative studies with more cases are needed.

In contrast, for patients in the rescue PDT group, the mean BCVA was maintained through the second year but tended to worsen gradually even though the mean CRT was maintained. More than half of patients maintained good VA at one year after the initial PDT treatment but only a small number of patients maintained good VA at five years. In total, 41% of patients in the initial PDT group did not require additional treatment within 1 year; however, 95% of the patients needed additional treatment during the follow-up period. The time until additional treatment was necessary was shorter in the rescue PDT group and the proportion of patients who required additional treatment during follow-up was also higher in the rescue PDT group. Regarding the additional treatment, at 12 months the mean numbers of anti-VEGF injections and PDT treatments were 1.9 and 0.15, respectively, and 6.7 and 0.32 (1.6 anti-VEGF injections annually) during the follow-up period. However, the average follow-up before the initial PDT application was 1.5 years and the mean number of anti-VEGF injections was 7.2 times (4.5 times annually). Therefore, the PDT may have reduced the necessity for anti-VEGF therapy. Wada et al. [21] retrospectively analyzed 23 patients with NV-AMD who underwent rescue PDT and reported that the number of anti-VEGF injections decreased from 5.8 times/year before rescue PDT to 2.6 times/year after PDT; central macular thickness improved from 319 µm to 226 µm and the VA changed from 0.16 to 0.11, which is not significant. Their results are similar to those of the current study as PDT decreased the number of additional treatments and improved the CRT but only maintained the VA. Liu and Chhabra [22] retrospectively analyzed the three-year outcomes of patients with PCV who had received combination therapy consisting of rescue PDT with either IVR or intravitreal aflibercept (IVA). They reported that the BCVA improved in all groups at different time points compared to the BCVA on the day that PDT was applied and the mean numbers of anti-VEGF injections required over 12, 24, and 36 months after PDT were all significantly lower than that those over the 12 months before PDT; it was pointed out that, in eyes refractory to IVR, performing rescue PDT promptly may be more beneficial than switching to IVA. Their results suggested that the timing of PDT should be discussed in the future.

In the current study, we also focused on the factors that ultimately preserve or worsen VA. A total of 18 of the 40 eyes that were followed for more than 5 years maintained good VA (0.22 or less logMAR unit). As reported previously [23], the presence of atrophy and fibrosis worsened the VA. Atrophy is a major factor in vision loss [23] and can occur with anti-VEGF therapy or PDT. However, it should be noted that, in this study, we examined the anatomic findings before treatment that may have affected the visual prognosis after PDT. HE and serous PED alone can cause poor vision but SRF, IRF, SRHM, and GLD by themselves are unlikely to cause poor vision; however, when combined with other findings, including HE and serous PED, these factors can cause poor vision. In addition, the longer the interval between PDT and the subsequent additional treatment, the better the visual prognosis tended to be. These results suggest that the findings before and during treatment initiation may predict the prognosis.

To minimize the damage to the choriocapillary bed caused by PDT, reduced-fluence (RF) PDT was used in combination with intravitreal anti-VEGF agents [18,24,25]. Sakurai et al. [18] compared IVR monotherapy to a combination of IVR and RF-PDT in eyes with PCV and reported that the combination of IVR and RF-PDT improved the BCVA as much as IVR monotherapy and required fewer additional IVR treatments. In contrast, Williams et al. [26] compared the combination of RF-PDT and ranibizumab to ranibizumab monotherapy for NV-AMD and reported no significant differences in VA, foveal thickness, or the number of additional IVR treatments. Recently, Ngo et al. [27] compared the efficacy of reduced- and standard-fluence PDT and reported no statistically significant difference between the retinal and choroidal anatomic OCT outcomes, rates of polyp closure, and recurrences between the two PDT regimens. Therefore, standard- and RF-PDT need to be examined with respect to the long-term prognosis of VA and the number of additional treatments. In our institution, STTA usually is used in combination with PDT to reduce the impact of PDT on the normal choroid and retina, except in patients with glaucoma, steroid responders, or patients at risk of cataract progression. STTA is often used in combination with PDT in Japan [19,20,28,29]. Hatta et al. [19] demonstrated that when combined with STTA, PDT therapy prolonged choroidal hypofluorescence by indocyanine green angiography compared with PDT monotherapy and may contribute to a reduction in the incidence and duration of the treatment. Ishikawa et al. [29] reported that the combination of STTA and PDT mitigated impaired retinal function soon after PDT and that retinal function was better preserved by that combination than by PDT monotherapy. Yoshizawa et al. [20] speculated that corticosteroids may suppress subretinal fibrosis that occurs after PDT by suppressing transforming growth factor-β. In contrast, no difference has been reported in the efficacy of STTA and PDT alone in the PCV [30]. Many reports do not provide adequate explanations and further studies are needed to clarify the mechanism of action and usefulness of STTA. Regarding the type of additional anti-VEGF therapy, IVR is often reported but there are also reports of combined IVA. Kikushima et al. [31] reported that, although there was no significant difference in the rate of retreatment between the therapeutic approaches during the 24-month follow-up period, PDT/IVA may be superior to PDT/IVR in terms of visual improvement. In addition to the previously used ranibizumab and aflibercept, brolucizumab [32] (Beovu^®^ Novartis, East Hanover, NJ, USA) and faricimab [33] (Vabysmo^®^ Roche, Basel, Switzerland) are currently approved in many countries to treat NV-AMD. Regarding brolucizumab, some reports have suggested that a high rate of complete regression of polypoidal lesions was achieved [34,35]. Also, it was suggested that brolucizumab improved the anatomic findings especially for obtaining a dry macula and stabilizing the BCVA and the number of injections needed in patients who have undergone previous treatment with other anti-VEGF agents [36,37]. Regarding faricimab, only short-term real-world outcomes have been reported [38,39]. The difference in the efficacy of anti-VEGF agents used for additional therapy remains to be investigated in future studies.

In recent years, the choroidal thickness has received much attention regarding AMD and related diseases. Pachychoroid is a relatively novel concept characterized by the attenuation of the choriocapillaris overlying dilated choroidal veins. The spectrum of pachychoroid disease includes central serous chorioretinopathy (CSC), pachychoroid pigment epitheliopathy, and pachychoroid neovasculopathy, which also includes PCV [40]. In CSC, PDT is also considered effective, with some reports focusing on the choroidal thickness before and after treatment [41,42,43]. In the present study, the choroidal thickness was not included in the evaluation because it was difficult to measure the choroidal thickness from OCT images as there were many cases before the measurement of choroidal thickness became common. In the future, we may predict cases for which PDT will be effective by investigating the relationship between PCV cases that maintain good VA after PDT and the choroidal thickness. Additionally, in recent years, the shortage of verteporfin has become an issue [44]. Although it did not affect the current study, it may affect future treatments with PDT.

The current study had several limitations, the first being its retrospective nature, along with the fact that there were no clear criteria for the initial treatment selection regarding whether PDT, anti-VEGF agents, or combination therapy including STTA should be used. The same was true for rescue PDT, as there were no criteria for the use of PDT. Secondly, the sample size was small. Thirdly, the results were not compared with patients who received anti-VEGF monotherapy during the study period at our institution. In addition, the difference between RF- PDT and standard-fluence PDT and the effect of STTA demand further study.

However, it is worth evaluating the long-term outcomes of patients treated with PDT following the approval of anti-VEGF agents. This study was retrospective and was conducted in the real world where the indication and treatment protocol was determined at the discretion of the physician and the study included patients with good VA and those who had already been treated with anti-VEGF therapy who were not included in the large prospective trial. Therefore, it is difficult to make a simple comparison but the average results are similar to those of EVEREST II [11,12], a large prospective study, and a meta-analysis in Japan [17]. However, it is important to note that, in the present study, PDT/STTA was effective in cases with pathological conditions similar to CSC, such as those without hard exudates, subretinal hemorrhages, or large polypoidal lesions, and that good long-term vision can be maintained with less treatment. In the future, it would be meaningful if PDT is also selected to treat PCV based on factors such as the choroidal thickness, size of the polypoidal lesions, and area of the branched vascular network.

## 5. Conclusions

Initial PDT application may be effective in about 30% of cases, requiring no additional therapy and preserving good vision. However, patients receiving rescue PDT need additional therapy in most cases and it may be difficult to maintain vision, nevertheless, PDT may preserve vision in some cases and reduce additional anti-VEGF therapy. In the future, it is necessary to consider the indication criteria for initial PDT and the timing of rescue PDT.

## Figures and Tables

**Table 1:** Patient characteristics.

**Initial PDT** **Group**	**35 Patients 38 Eyes**	
Age		69.1 ± 11.1 years
Follow-up period		6.2 ± 2.64 years
GLD		3047 ± 1223 µm
Treatment regimen for initial PDT	PDT + STTA	29 eyes (76%)
	PDT + STTA + anti-VEGF	5 eyes (13%)
	PDT	4 eyes (11%)
**Rescue PDT Group**	**22 Patients 22 Eyes**	
Age		69.8 ± 8.7 years
Follow-up period		5.8 ± 2.7 years
GLD		3200 ± 1110 µm
Previous VEGF inhibitor therapy		7.3 ± 6.3 times
Time from initial treatment		1.6 ± 1.5 years
Follow-up period after initial PDT		4.3 ± 2.5 years
Previous treatment regimen with anti-VEGF	PRN	19 eyes (86%)
	TAE	2 eyes (9%)
	PRN switched to TAE	1 eye (5%)
Reasons for rescue PDT	Frequent recurrences	13 eyes (59%)
	Resistance to treatment	5 eyes (23%)
	Patient request	4 eyes (18%)
Treatment regimen for rescue PDT	PDT + STTA	14 eyes (64%)
	PDT + STTA + anti-VEGF	8 eyes (36%)

**Table 2:** Additional treatment after the first PDT treatment.

	Period	Treated Eyes	Additional Treatment and Numbers	
Initial PDT group (38 eyes)	Within 1 year	17 eyes (45%)	Anti-VEGF (12 eyes)	2.3 ± 1.2 times
			PDT + STTA (4 eyes)	1.0 ± 0 times
			PDT + STTA + anti-VEGF (1 eye)	1 (PDT)/1 (anti-VEGF) times
	During follow-up period (mean, 6.2 years)	27 eyes (71%)	Anti-VEGF (21 eyes)	7.5 ± 6.7 times
			PDT + STTA (3 eyes)	1.0 ± 0 times
			PDT + STTA + anti-VEGF (3 eyes)	1.3 ± 0.5 (PDT)/18.3 ± 15.5 (anti-VEGF) times
Rescue PDT group (22 eyes)	Within 1 year	13 eyes (59%)	Anti-VEGF (11 eyes)	1.9 ± 1.3 times
			PDT + STTA (0 eyes)	
			PDT + STTA + anti-VEGF (2 eyes)	1.0 ± 0 (PDT)/1.0 ± 0 (anti-VEGF) times
	During follow-up period (mean, 4.3 years)	21 eyes (95%)	Anti-VEGF (16 eyes)	7.0 ± 6.0 times
			PDT + STTA (0 eyes)	
			PDT + STTA + anti-VEGF (5 eyes)	1.4 ± 0.8 (PDT)/5.8 ± 2.8 (anti-VEGF) times

All additional PDT applications were performed in combination with STTA.

**Table 3:** Correlations between final VA and parameters.

		rs	*p*
Time until additional treatment		−0.260	0.045
Anatomic findings before treatment	SRF	−0.075 ^a^	0.569
	IRF	0.048 ^a^	0.714
	Serous PED	0.322 ^a^	0.012
	SRH	−0.061 ^a^	0.643
	HE	0.263 ^a^	0.042
	SHRM	0.092 ^a^	0.483
	GLD	0.131 ^a^	0.318
	Vision-threatening score	0.273 ^a^	0.035
BCVA at baseline		0.277	0.032
BCVA at year 1		0.758	0.000
Anatomic findings at final visit	Fibrosis	0.286 ^a^	0.026
	Atrophy	0.358 ^a^	0.005

rs, Pearson’s correlation coefficient; ^a^ rs, Spearman’s rank correlation coefficient.

## Data Availability

Researchers can contact Aki Kato, (akikato@med.nagoya-cu.ac.jp) for details of the protocol and results.
